# The Environment of Birthplace and Self-Reported Mental Health Conditions: Findings from the American Panel of Life

**DOI:** 10.3390/epidemiologia2030019

**Published:** 2021-07-12

**Authors:** Hans Oh, Jessica Goehring, Louis Jacob, Lee Smith

**Affiliations:** 1 Suzanne Dworak-Peck School of Social Work, University of Southern California, Los Angeles, CA 90015, USA; jgoehrin@usc.edu; 2 Research and Development Unit Parc Sanitari Sant Joan de Déu, CIBERSAM, 08839 Barcelona, Spain; louis.jacob.contacts@gmail.com; 3 Faculty of Medicine, University of Versailles Saint-Quentin-en-Yvelines, 78180 Montigny-le-Bretonneux, France; 4 Centre for Health, Performance and Wellbeing, Anglia Ruskin University, Cambridge CB1 1PT, UK; Lee.Smith@aru.ac.uk

**Keywords:** urban, rural, psychiatric disorders, city, suburb, metropolitan

## Abstract

Studies from around the globe have found that urbanicity is associated with greater risk for certain psychiatric disorders, though the association has been less evident in the United States. We analyzed data collected in 2019 from the RAND American Life Panel (*n* = 2554), which were representative of the general adult population of the United States. Using multivariable logistic regression, we examined the associations between environment of birthplace (large urban, small urban, suburban, rural) and psychiatric disorders, adjusting for sociodemographic characteristics. We found that being born in a large urban area was associated with greater odds of having any psychiatric disorder when compared with being born in a rural area. However, when looking at specific disorders, we found that being born in a large urban area was only significantly associated greater odds of anxiety disorder and post-traumatic stress disorder (PTSD), but was not associated with bipolar disorder, major depressive disorder, attention deficit/hyperactivity disorder, or alcohol/substance use disorder. Being born in a small urban area was marginally associated with anxiety disorder. Future studies should examine why urban birthplace has only been associated with anxiety disorders and PTSD in the United States, and why urbanicity is associated with mood disorders in other parts of the world but not in the United States.

## 1. Introduction

Over the past century, the United States has become increasingly urbanized, which may have contributed to the development of mental health problems for population. While the definition of urbanicity has lacked consistency in research [1], we define “urban” as cities and surrounding areas that are characterized by high density (e.g., at least 2500 inhabitants), large size, and high levels of population heterogeneity (see the early work of [2]. Internationally, urbanization has been linked to several mental health problems, attributed to social disparities (see [1]), violence exposure [3], adversities and traumas [4], pollution [5], and absence of natural environments or green spaces [6]. A meta-analysis found that psychiatric disorders (particularly mood and anxiety disorders) were more prevalent in urban areas when compared with rural areas, though this was not the case for substance use disorders [7]. However, in the United States, the association between urban living and psychiatric disorders has been unclear. For example, one study that used data from the National Comorbidity Survey Replication (NCS-R) showed that while drug abuse was more common among people currently residing in urban areas when compared with people residing in non-urban (i.e., suburban or rural) areas, rurality was not associated with risk for any other lifetime psychiatric disorders [4]. Another study using the NCS-R found that growing up in a large city was not significantly associated with any psychiatric disorders for White respondents; on the other hand, data from the National Survey of American Life showed that growing up in a large city was significantly associated with greater odds of having mood disorder, and growing up in a non-rural area was associated with greater odds of alcohol and substance use disorders in a representative sample of Black Americans [8]. Finally, a study using the National Health and Nutrition Examination Survey data found no differences in risk between the most rural areas and the largest urban areas [9]. In light of these mixed findings, we sought to re-examine the associations between environment of birthplace and self-reported lifetime psychiatric disorders in the United States. 

## 2. Methods

### 2.1. Sample

This study analyzed data from the RAND American Life Panel (ALP) [10], which is a US nationally representative probability-based panel. All panel members were over the age of 17. Approximately 6000 panel members were recruited using probability sampling methods (telephone random-digit dial samples and address-based samples). Panel members without computers or internet were provided these resources in order to enhance representativeness of the panel. In 2019, RAND administered (1) the ALP Omnibus Survey (*n* = 2555) conducted in February–April; and (2) the Health and Functional Capacity Survey (*n* = 2657) conducted April–June. The response rates were 64.9% and 78.2%, respectively. All of the panel members who completed the ALP Omnibus Survey also received the Health and Functional Capacity Survey; thus, we merged the two data sets, yielding a final analytic sample of *n* = 2554 for the current study. RAND constructed sampling weights to correct for sampling error and to make the sample as representative of the general population as possible using benchmark distributions derived from the Current Population Survey. Weights were created using a raking process, accounting for gender, number of household members, race/ethnicity, education level, income, and other characteristics. All data collection and survey protocols were approved by RAND’s Human Subjects Protection Committee, which serves as RAND’s Institutional Review Board.

### 2.2. Measures

#### 2.2.1. Environment of Birthplace (Independent)

The environment of upbringing was measured using a single item: “Describe the area in which you were born.” Respondents could answer large urban (>500,000 people), small urban (<500,000 people), suburban, or rural. 

#### 2.2.2. Psychiatric and Substance Use Disorders (Dependent)

Psychiatric disorders were self-reported and coded dichotomously to reflect the presence of the following conditions: bipolar disorder, major depressive disorder, anxiety disorder, attention deficit hyperactivity disorder (ADHD), Post-Traumatic Stress Disorder (PTSD), and substance use disorders (any alcohol dependence, opioid dependence, other substance use disorder).

#### 2.2.3. Sociodemographic Characteristics (Covariates)

Sociodemographic covariates included age (continuous), sex (male, female), race/ethnicity (non-Hispanic Black, non-Hispanic White, Hispanic/Latinx, Other), income (less than USD 25,000, USD 25,000–49,999, USD 50,0000–74,999, USD 75,000–99,999, USD 100,000–124,999, USD 125,000–199,999, USD 200,000 or more), education (less than high school, some high school but no diploma, high school graduate or equivalent, some college but no degree, professional school degree, Associate’s degree, Bachelor’s degree, Master’s degree, Doctoral degree), immigrant status (US-born/foreign-born), and health insurance coverage (yes/no). 

### 2.3. Analysis

We first examined the prevalence of psychiatric disorders across environments of upbringing. We then ran bivariate logistic regression models examining the associations between environment of birthplace and each individual psychiatric disorder (Supplemental Materials Table S1). Using multivariable logistic regression, we examined environment of birthplace and psychiatric disorders, adjusting for sociodemographic characteristics. We reported effects sizes as odds ratios with 95% confidence intervals (a = 0.05). 

## 3. Results

**Figure 1:**
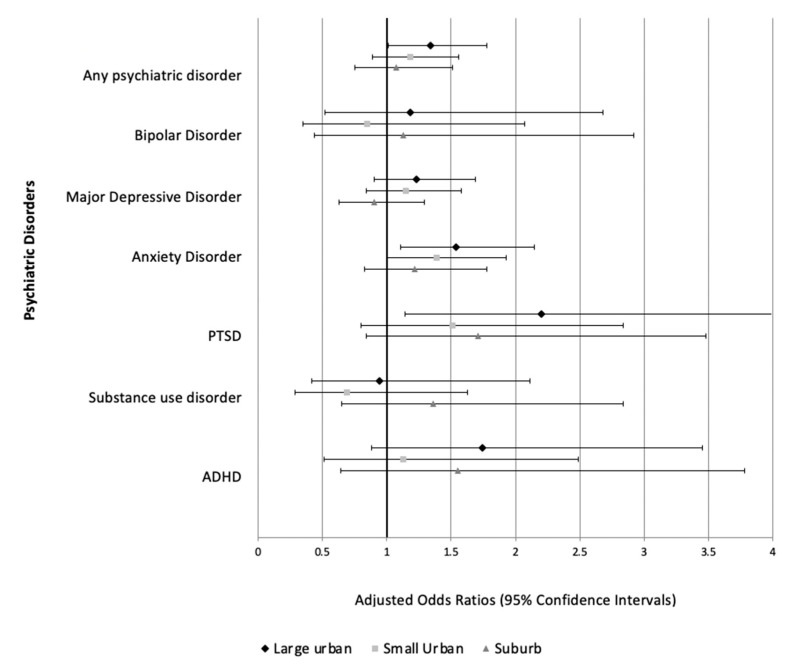
Multivariable logistic regression models showing associations between environment of birthplace and psychiatric disorders. (Reference group = Rural).

We found that being born in a large urban area was associated with 1.40 times greater odds of having any psychiatric disorder when compared with being born in a rural area. This association attenuated but remained significant after adjusting for age, sex, education, household income, race/ethnicity, and insurance status (aOR: 1.34; 95% CI: 1.01–1.78). However, when looking at specific disorders in fully adjusted models, we found that being born in a large urban area was only significantly associated greater odds of anxiety disorder (aOR: 1.54; 95% CI: 1.11–2.15) and PTSD (aOR: 2.20 95% CI: 1.14–4.24), but was not associated with bipolar disorder, depression, ADHD, or substance use disorder. Being born in a small urban area was marginally associated with anxiety disorder (aOR: 1.39 95% CI: 1.00–1.93) (Figure 1). All multivariable logistic regression models can be found in the Supplemental Materials Table S1.

## 4. Discussion

### 4.1. Main Findings

Based on a nationally representative sample of the US general adult population, being born in a large urban area was significantly associated with any self-reported psychiatric disorder, when compared with being born in a rural area, adjusting for sociodemographic covariates. However, when examining specific conditions, being born in a large urban area was only associated with greater odds of having self-reported anxiety disorder and PTSD and being born in a small urban area was marginally associated with greater odds of anxiety disorder. Our study’s findings diverge from prior studies, though we only examined environment of birthplace (rather than environment of upbringing or current residence), which may explain some of the discrepancies. At the same time, our study contributes to the literature by focusing on early and sensitive periods of the life course during which individuals may be particularly sensitive to environmental exposures that affect psychological development. Scholars have hypothesized that the prevalence of psychiatric disorders is higher in urban areas due to the higher levels of environmental insults, such as pollution [11,12], noise [13], and psychosocial stressors (e.g., adversities, traumatic experiences, victimization, violence, discrimination [4]), which seems to align with our findings, though we cannot reconcile why urban birthplace was not associated with mood or substance use disorders. 

### 4.2. Limitations and Future Directions

Our findings should be interpreted in light of a number of limitations. First, the data were cross sectional and did not allow for us to make any causal inferences or understand the dynamic interplay between place and mental health over time. Interdisciplinary methods using experimental designs, ecological momentary assessments, and ethnographies may elucidate the nexus between urban environments and mental health (see [14]). Second, while the sample was nationally representative, it may have been vulnerable to selection bias. For example, the survey was only administered in English to individuals who have residential addresses and may have therefore excluded immigrants with limited English proficiency or people affected by homelessness. Third, the measure for psychiatric disorders was based on self-report, which is subject to recall and social desirability biases. Analyzing administrative data (i.e., medical records, insurance claims) may circumvent some of these threats to validity, though not entirely. It is also possible that mental health services are less accessible to poor rural residents (due to proximity of clinics/hospitals and transportation problems, the cost of care, and stigma) [15], and without access to mental health services and psychiatric providers, rural residents may have had undiagnosed disorders. Future studies can improve upon the findings by using lay-administered structured interviews to more accurately assess the presence of psychiatric disorders. Fourth, respondents self-reported their environment of birthplace, which may have resulted in some error given that the definition of ‘urban’ or ‘rural’ is somewhat relative and open to interpretation for respondents. Future studies can use objective measures of urbanicity using addresses/zip codes to precisely locate and ascertain the urbanicity of the environments in which respondents were born and raised. Fifth, while we differentiated between large urban areas, small urban areas, suburban, and rural areas, we did not differentiate between rural and semi-rural areas. Breslau and colleagues found risk for major depression and other serious mental illnesses was higher in the semi-rural areas (e.g., towns) than in the large urban areas, calling for future studies to distinguish semi-rural and rural areas into two categories. Sixth, associations between environment of birthplace and mental health outcomes may have not achieve a conventional level of statistical significance because analyses may have been under-powered. For example, very few respondents reported substance use, which did not allow for a more nuanced exploration of how environment of birthplace might be related to specific drug use. Seventh, it is possible that despite the sampling strategy and survey weights employed, the data may have undercounted psychiatric disorders. While approximately 27% of the RAND ALP analytic sample reported any psychiatric disorder, this figure more closely resembles the proportion of the general population who experiences mental illness over the past year (around 20%) rather than lifetime prevalence (50%) (see [16]). Finally, we assumed that environment of birthplace was also an indication of the environment of upbringing; however, it is possible that families may have moved shortly after the respondent was born, such that birthplace does not necessarily reflect where respondents were raised. Thus, future studies can take into account the rate of residential mobility, where families move to different environments throughout the respondent’s childhood and adolescence.

### 4.3. Conclusions

Urban birthplace was significantly associated with any psychiatric disorders, though when disaggregating the conditions, was only associated with anxiety disorders and PTSD. An open area of research is to explore why urbanicity was not associated with mood disorders and other disorders in the United States even when such an association is found in other parts of the world.

## Data Availability

Public versions of RAND American Life Panel data (de-identified) are available at https://www.rand.org/research/data/alp.html (accessed on 1 January 2021).

## Supplementary Materials

The following are available online at https://www.mdpi.com/article/10.3390/epidemiologia2030019/s1, Table S1. Associations between environment of birthplace and psychiatric disorders.
