# Supplementary material for: The Environment of Birthplace and Self-Reported Mental Health Conditions: Findings from the American Panel of Life
Source: Epidemiologia (Basel). 2021 Jul 12;2(3):256–61. doi: 10.3390/epidemiologia2030019 (PMC9620909; doi:10.3390/epidemiologia2030019)
Supplement: Supplementary file 1 [file epidemiologia-02-00019-s001.zip › epidemiologia-1287193-supplementary.pdf]

Supplementary Materials: The Environment of Birthplace and Self-Reported Mental Health Conditions: Findings from the American Panel of Life

Hans Oh, Jessica Goehring and Louis Jacob and Lee Smith

Table S1. Associations between environment of birthplace and psychiatric disorders.

**Associations between environment of birthplace and psychiatric disorders**					
**Any Mental Health Disorder**					
		Unadjusted		Adjusted	
Environment of birthplace	n/N	OR [95% CI]	P-value	aOR[95% CI]	*P*-value
Big city	232/776	**1.40 [1.08–1.82]**	**0.01**	**1.34 [1.01–1.78]**	**0.04**
Small city	187/678	1.25 [0.96–1.63]	0.10	1.18 [0.89–1.56]	0.26
Suburb	111/443	1.10 [0.79–1.53]	0.57	1.07 [0.75–1.51]	0.71
Rural	125/536	1.00	-	1.00	-
**Bipolar disorder**					
		Unadjusted		Adjusted	
Environment of birthplace	n/N	OR [95% CI]	P-value	aOR[95% CI]	*P*-value
Big city	23/776	1.61 [0.74–3.47]	0.23	1.18 [0.52–2.68]	0.68
Small city	13/678	1.03 [0.45–2.37]	0.95	0.85 [0.35–2.07]	0.72
Suburb	10/443	1.21 [0.50–2.97]	0.67	1.13 [0.44–2.92]	0.80
Rural	10/536	1.00	-	1.00	-
**Major Depressive Disorder**					
		Unadjusted		Adjusted	
Environment of birthplace	n/N	OR [95% CI]	P-value	aOR [95% CI]	*P*-value
Big city	153/176	1.29 [0.95–1.74]	0.10	1.23 [0.90–1.69]	0.19
Small city	127/678	1.21 [0.89–1.63]	0.22	1.15 [0.84–1.58]	0.38
Suburb	66/443	0.92 [0.66–1.28]	0.61	0.90 [0.63–1.29]	0.57
Rural	86/536	1.00	-	1.00	-
**Anxiety Disorder**					
		Unadjusted		Adjusted	
Environment of birthplace	n/N	OR [95% CI]	P-value	aOR	*P*-value
Big city	161/776	**1.63 [1.20–2.23]**	**0.00**	**1.54 [1.11–2.15]**	**0.01**
Small city	132/678	**1.51 [1.10–2.06]**	**0.01**	1.39 [1.00–1.93]	0.05
Suburb	75/443	1.27 [0.88–1.83]	0.20	1.22 [0.83–1.78]	0.31
Rural	74/536	1.00	-	1.00	-
**Post Traumatic Stress Disorder**					
		Unadjusted		Adjusted	

Environment of birthplace	n/N	OR [95% CI]	P-value	aOR[95% CI]		*P*-value
Big city	48/776	**2.29 [1.23–4.27]**	**0.01**	**2.20 [1.14–4.24]**		**0.02**
Small city	32/678	1.72 [0.92–3.21]	0.09	1.51 [0.80–2.84]		0.20
Suburb	21/443	1.73 [0.87–3.43]	0.12	1.71 [0.84–3.48]		0.14
Rural	15/536	1.00	-	1.00		-

Any substance use disorder

		Unadjusted		Adjusted	
Environment of birthplace	n/N	OR [95% CI]	*P*-value	aOR[95% CI]	*P*-value
Big city	16/776	0.92 [0.43–1.99]	0.83	0.94 [0.42–2.11]	0.88
Small city	11/678	0.72 [0.31–1.66]	0.44	0.69 [0.29–1.63]	0.39
Suburb	13/443	1.32 [0.60–2.88]	0.48	1.36 [0.65–2.84]	0.41
Rural	12/536	1.00	-	1.00	-

Attention Deficit Hyperactivity Disorder

		Unadjusted		Adjusted	
Environment of birth-place	n/N	OR [95% CI]	P-value	aOR[95% CI]	*P*-value
Big city	29/776	1.85 [0.96–3.59]	0.07	1.74 [0.88–3.45]	0.11
Small city	17/678	1.23 [0.57–2.65]	0.60	1.13 [0.51–2.49]	0.76
Suburb	14/443	1.56 [0.66–3.69]	0.31	1.55 [0.64–3.78]	0.33
Rural	11/536	1.00	-	1.00	-

aOR (95% CI), $p < 0.05$ indicated in bold
